# Discoid lupus erythematosus successfully treated with deucravacitinib

**DOI:** 10.1016/j.jdcr.2024.04.032

**Published:** 2024-04-30

**Authors:** Nnenna Ezeh, Ruth Ann Vleugels, Neda Shahriari

**Affiliations:** a Harvard Combined Dermatology Residency, Massachusetts General Hospital, Boston, Massachusetts; b Department of Dermatology, Brigham and Women's Hospital, Harvard Medical School, Boston, Massachusetts

**Keywords:** cutaneous lupus erythematosus, deucravacitinib, discoid lupus erythematosus, emerging treatments, interferon, TYK-2 inhibitor

## Introduction

Discoid lupus erythematosus (DLE) is a scarring form of cutaneous lupus erythematosus (CLE) characterized by inflammation, dyspigmentation, and alopecia. Given the degree of disfigurement it causes, there is significant negative impact on quality of life. Management of DLE has largely consisted of antimalarials and topical/intralesional corticosteroids. Mycophenolate mofetil, methotrexate, and thalidomide/lenalidomide have been utilized as second and/or third line agents with more recent studies supporting the use of anifrolumab in recalcitrant DLE cases.^1^ Deucravacitinib is an oral, first-in-class, selective tyrosine kinase-2 (TYK-2) inhibitor and member of the Janus kinase family approved for the treatment of moderate-to-severe plaque psoriasis, and, in Japan, also for pustular and erythrodermic psoriasis.^2^^,^^3^ A recent phase II clinical trial demonstrated promise for the management of systemic lupus erythematosus (SLE)^4^ while few case reports have shown improvement in tumid lupus^5^ and subacute cutaneous lupus erythematosus.^6^^,^^7^ We report a case of a patient with concomitant DLE and palmoplantar pustular psoriasis who had rapid improvement of her DLE on deucravacitinib.

## Case report

**Fig 1:**
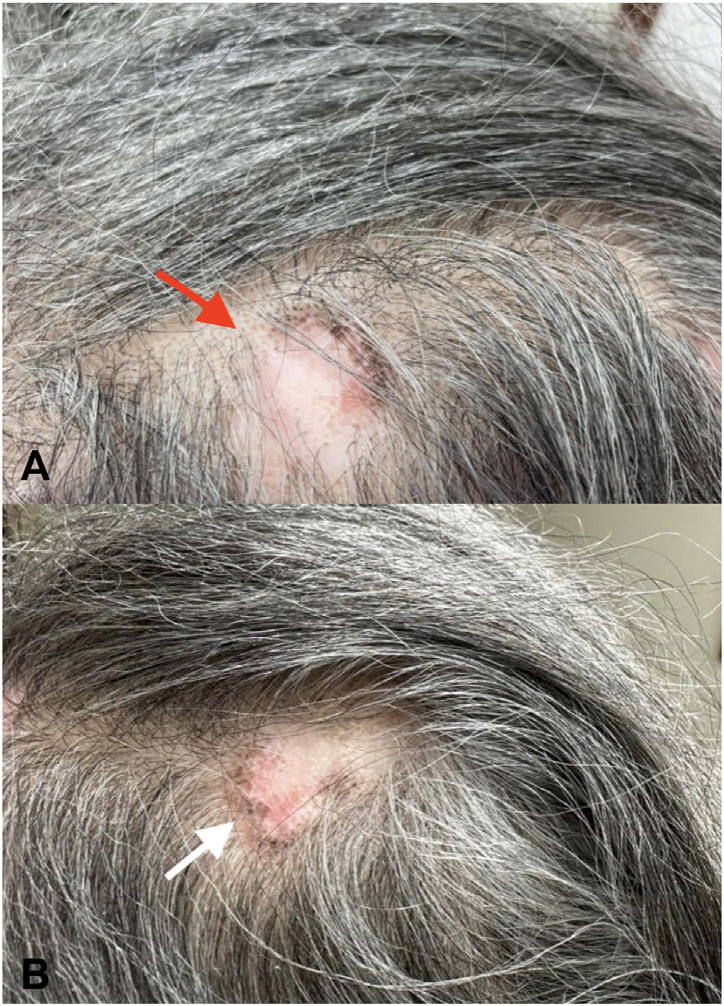
Clinical findings prior to treatment. Multiple quarter-sized plaques of alopecia with areas of hypopigmentation, scaly erythema with follicular keratotic plugs, and peripheral hyperpigmentation on the scalp indicative of active inflammation. **A,** Left frontal parietal scalp lesion (*red arrow*). **B,** Left posterior parietal scalp lesion (*white arrow*).

A 65-year-old woman was referred to our clinic for management of biopsy-proven DLE of the scalp and ears diagnosed in 1991. She had been managed on hydroxychloroquine 200 mg twice daily and fluocinolone oil as needed along with intralesional triamcinolone. Despite this regimen, she reported recent increase in areas of hair loss as well as scalp pain and pruritus concerning for active disease. An evaluation of her scalp revealed multiple quarter-sized plaques of alopecia with areas of hypopigmentation, scaly erythema with follicular keratotic plugs, and peripheral hyperpigmentation (Fig 1). Adjuvant methotrexate and acitretin were previously attempted without improvement. Topical betamethasone dipropionate was minimally effective.

Patient denied any clinical symptoms of SLE including oral ulcers, arthritis, pleurisy, and constitutional and neurologic symptoms. Further workup included antinuclear, double-stranded DNA, and anti-Smith antibodies, as well as complements C3 and C4, complete blood count with differential, and comprehensive metabolic panel, which were all within normal limits. Therefore, no concerns were noted for SLE in this patient.

**Fig 2:**
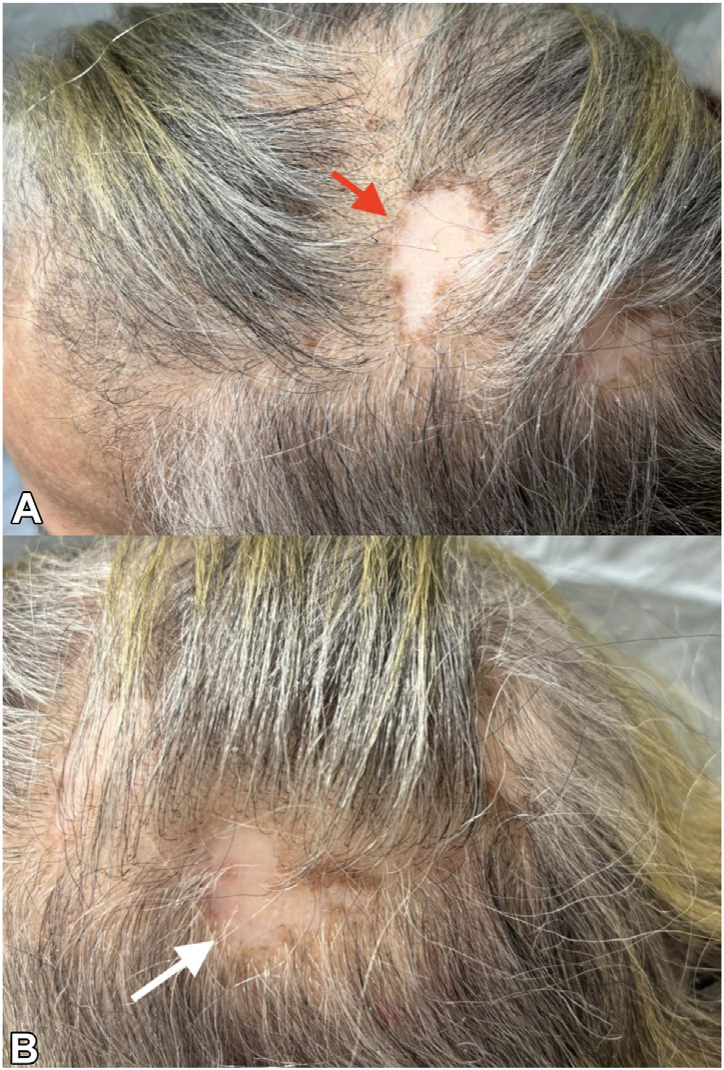
Clinical findings 1 month after initiation of deucravacitinib. There is clearance of scalp erythema and follicular keratotic plugs indicating resolution of inflammation. **A,** Left frontal parietal scalp lesion (*red arrow*). **B,** left posterior parietal scalp lesion (*white arrow*).

In addition to her DLE, patient presented with palmoplantar pustular psoriasis present for 6 months. On exam, scattered pustules were noted on the palms, while on the soles, psoriasiform plaques studded with pustules were observed. A potassium hydroxide prep test was previously performed which was negative. Patient had also failed treatment with topical ketoconazole 2% cream, oral terbinafine, topical tacrolimus, and betamethasone dipropionate. Deucravacitinib 6 mg daily was initiated in addition to continuing hydroxychloroquine 200 mg twice daily. After 1 month of therapy, the patient noted disease remission of DLE with resolution of scalp pain, scalp pruritus, erythema, and follicular keratotic plugs (Fig 2). Follicular keratotic plugs have previously been shown to signify active disease.^8^ In addition, there was significant flattening and reduction in the size of many palmoplantar pustular lesions. A mild inflammatory acneiform eruption was noted on the face secondary to deucravacitinib, which is being managed with topical clindamycin.

## Discussion

Treatment of DLE can be difficult given the recalcitrant nature of disease. In this case, a secondary goal was to identify a therapy that could potentially treat 2 conditions—palmoplantar pustular psoriasis and DLE—to avoid polypharmacy.

To our knowledge, there have been no reports regarding the use of deucravacitinib in DLE, specifically. Possible efficacy of deucravacitinib was raised in case reports involving patients with subacute cutaneous lupus erythematosus and tumid lupus.^5^, ^6^, ^7^ We present the first case to date of the use of deucravacitinib for biopsy-proven DLE. Our patient achieved rapid improvement in her symptoms of active DLE in just 1 month, indicating a potential therapeutic role of TYK-2 inhibitors in DLE management. Her palmoplantar pustular psoriasis also improved on deucravacitinib.

Recently, there has been promising data for TYK-2 inhibitors in the management of lupus erythematosus. TYK-2 has been previously noted as a susceptibility gene in SLE with studies showing it confers risk for DLE as well.^9^ More specifically, TYK-2 can transduce signaling from various cytokines, including interleukin (IL)-10, IL-12, IL-23, and type 1 interferon, which importantly, IL-12 and type 1 interferon signaling have been implicated in SLE and DLE.^10^ In a 48-week phase II clinical trial assessing deucravacitinib for SLE, a secondary outcome was evaluation of the Cutaneous Lupus Erythematosus Disease Area and Severity index-50 response scores.^4^ As measured by Cutaneous Lupus Erythematosus Disease Area and Severity index-50 response, nearly 70% of patients saw 50% or more improvement in the treatment group receiving deucravacitinib compared to just 17% of patients in the placebo group.^4^ Notably, this was a generalized cutaneous endpoint without specific CLE subtype analysis, prompting the need for further cutaneous specific studies. The most common adverse events reported in the study included upper respiratory tract infections, urinary tract infections, and nasopharyngitis.^4^ Only 3.3% of patients developed acne on the 6-mg dose, which was an adverse event noted in our patient.

In summary, we report the successful use of deucravacitinib in the treatment of DLE and support preliminary data regarding the efficacy of TYK-2 inhibitors in the treatment of the CLE spectrum. To our knowledge, this is the first report of the use of deucravacitinib specifically for DLE and suggests that it may serve as a promising therapeutic option in this CLE subtype. Additionally, for our patient, we were able to utilize 1 targeted treatment option to improve both DLE and palmoplantar pustular psoriasis, highlighting the importance of treatment options that can help avoid polypharmacy. Further cases are needed to better elucidate the impact of TYK-2 inhibitors in managing DLE as well as other CLE subtypes.

## Conflicts of interest

None disclosed.
